# Health worries, life satisfaction, and social well-being concerns during the COVID-19 pandemic: Insights from Lebanon

**DOI:** 10.1371/journal.pone.0254989

**Published:** 2021-07-29

**Authors:** Imad Bou-Hamad, Reem Hoteit, Dunia Harajli

**Affiliations:** 1 Department of Business Information and Decision Systems, Suliman S. Olayan School of Business, American University of Beirut, Beirut, Lebanon; 2 Department of Epidemiology and Population Health, Faculty of Health Sciences, American University of Beirut, Beirut, Lebanon; 3 Department of Information Technology and Operations Management, Adnan Kassar School of Business, Lebanese American University, Beirut, Lebanon; College of Medicine and Sagore Dutta Hospital, INDIA

## Abstract

The COVID-19 outbreak has struck Lebanon in its worst period of instability, not only impacting physical health, but also increasing psychological distress. Using an online survey enhanced by response time measurement, this study describes the overall patterns in mental well-being outcomes and examines their association with sociodemographic characteristics during the COVID-19 pandemic. Furthermore, it identifies significant predictors for COVID-19 good practices. A total of 988 Lebanese were surveyed, with participants providing written online consent prior to filling the survey. Regression-based models were estimated. Findings show that individuals with higher education levels exhibit lower health concerns. People with children face higher health worries than those without. Men are more worried than women about their health and they are less satisfied with their lives during the pandemic. Descriptive statistics show that most Lebanese are very satisfied with their families (93.1%), but they are highly dissatisfied with their country (63%). Young adults and individuals who live alone exhibit significantly higher social well-being concerns. Age and having children were strong predictors for good COVID-19 practices. The odds of having good practices for older adults are 3.13 times higher than that of youth, while the odds for those with children are 3.18 times higher than those without. The findings of this study could pave the way for a well-coordinated national strategy and increased collaboration with public health professionals to mitigate the pandemic’s adverse effects on mental health in the long-term.

## Introduction

Corona Virus Disease (COVID-19) is highly infectious and is caused by *Severe Acute Respiratory Syndrome Coronavirus 2* (Sars-Cov-22), which has a long incubation period. COVID-19 has had an unexpected and tremendous influence on populations across the world. Consequently, preventive actions have been imposed to minimize physical distance to reduce the spread of the virus. Classrooms, childcare, community facilities and workplaces were therefore forced to abruptly close, leaving many people across the globe unemployed [1]. The implications of these changes are having an important effect not only on the financial status (e.g., financial insecurity) [2], but also on the population’s psychological health and well-being (e.g., loneliness, depression, anxiety and life satisfaction) [3].

Stress, anxiety, depression, and many mental health issues have spread among societies across all continents. Studies in the literature showed that job loss, financial burden, and social isolation were linked to mental health disorders [4–6]. Data from the United Kingdom National Statistics Office (ONS) revealed that about 72% of the UK population were concerned about the effects of COVID-19 on their life, many showed a higher degree of anxiety (32%), a disrupted well-being (43%) and loneliness (23%) [7]. In two studies carried out in China, Chinese respondents expressed health worries about themselves and their families being infected with COVID-19 [8, 9].

In addition, Rossell et al. showed that there was a high level of negative emotions (depression, tension, and anxiety) among Australians individuals [10]. Another study conducted in China found that negative emotion cues and perceived social risk increased significantly, while positive emotion cues such as hope, happiness, and life satisfaction decreased when compared to those before the pandemic [11].

Furthermore, according to a recent systematic review analyzing the effect of the COVID-19 pandemic on mental health, the prevalence of signs of adverse mental results in the population is higher relative to prevalence before the pandemic [12]. The same research conducted by Xiong et al. found that symptoms of anxiety varied from 6.33% to 50.9%. Interestingly, this study has also found that more adverse psychiatric symptoms were repeatedly identified in the female gender, younger age group (≤ 40 years), and student population. Other studies have also indicated that lower education level, poor economic status and unemployment are important risk factors for the development of symptoms of mental disorders during the pandemic period [12–14].

While herd immunity against COVID-19 has yet to be achieved, good practices by the local population remain essential for controlling the virus’s spread. Based on the results of a systematic review and meta-analysis conducted by Bhagavathula et al., a total of 73 percent (95% CI: 61–85) stated a practice related to wearing a face mask to prevent the transmission of COVID-19 [15]. Furthermore, the same systematic study found that 82 percent (95% CI: 68–95) of people practiced hand hygiene to avoid contracting COVID-19. A recent study conducted in Lebanon showed that most participants demonstrated proactive practices to protect themselves against COVID-19 such as covering their mouths (81.2%), discarding the used tissues (93.7%) and washing their hands after sneezing or coughing (66.6%) [16].

### Background on Lebanon

Lebanon is a middle-income country in the Middle East with a long history of complicated political instability and conflict, where civil war, border disputes, war with neighboring countries, and conflict in Syria have all had a profound effect on this country and its health system [17]. Following the outbreak of a nationwide revolution in October 2019, political and economic climates have seen Lebanon reach its worst point in decades, and perhaps since its independence in 1943. And recently, this nation has been struggling with a severe economic recession, poverty and rapid currency devaluation crisis [18, 19]. Needless to say, such a load of stressors can take its toll on a population, even one as resilient as the Lebanese [20].

For decades, the Lebanese population has suffered an accumulation of psychological trauma [21]. A national survey conducted in Lebanon prior to the Syrian conflict found that one in six individuals met the requirements for at least one mental disorder, with 27.0% of them identified as "serious" [22]. Additionally, a high prevalence of mental health outcomes in populations facing cluster munitions has been identified in numerous other studies conducted in Lebanon (post-traumatic stress disorder 98% in 2006 and 43% in 2016) [23].

Since late 2019, the world has struggled to deal with the COVID-19 outbreak and the first case was confirmed in Lebanon on February 21, 2020. The Government of Lebanon introduced a full lockdown on March 18, 2020 in an attempt to flatten the curve, granting the authorities a legal mandate to implement special measures against COVID-19, including closures of borders (airport, sea and land) and public and private establishments [21, 24]. This has contributed to many challenges, including increased fear of infection, dramatic lifestyle changes due to lockdown measures, and increased unemployment levels, thereby impacting the population’s psychological well-being and mental health [21].

To the best of our knowledge, no sufficient research has been conducted in Lebanon to assess health worries, social well-being, and life satisfaction during the COVID-19 outbreak. The aim of the present research is therefore to describe the overall patterns in mental well-being outcomes and investigates their association with sociodemographic characteristics during the COVID-19 pandemic. In addition, we explore predictors that influence good practice in reducing the spread of the COVID-19 virus.

## Methods

### Study design and data collection

The COVID-19 pandemic prompted academics and several research institutes to conduct studies to better understand the virus’s effects from various angles. A global research and technology provider launched an international COVID-19-related project involving 20 countries, including Lebanon. The project aimed initially at understanding people’s emotions, fears, behaviors, and perspectives on the pandemic in different countries. More specifically, this global project was supposed to cover the aspects of functioning most affected by the pandemic, such as lifestyle changes (isolation, following guidelines, etc.), economic threats (losing jobs, not earning money due to lockdown), and emotional stress (especially health worries).

The data collection was carried out through an online survey using iCode [25, 26] smart test. This test illustrates how hesitant people are to express their views and is a simpler alternative to Implicit Association Testing (IAT), where IAT is commonly used in psychology [27]. More specifically, iCode uses reaction time testing to measure people’s responses to questions by recording the speed and the rhythm with which the screen is touched. Reported opinions with a relatively quick reaction time are expected to reflect actual behavior [28].

The questionnaire was created and validated with the help of eight experts from the fields of psychology, sociology, marketing, and economics. It was a collaborative effort involving experts from the United States, Poland, Singapore, Hong Kong, Portugal, and Switzerland.

The questionnaire was distributed in Lebanon in three languages (Arabic, English, and French) and a sample of 988 Lebanese respondents was collected between May 3 and May 31 of 2020. Prior to filling the survey, all participants provided written informed consent online. Due to the pandemic’s rapidly changing context and to minimize the virus’s spread, we used an online convenience sampling approach. This sampling technique has been widely used in many COVID-19-related studies [16, 29–31]. A link for the survey with a description, was distributed via social media platforms, including WhatsApp, Instagram, Facebook, and LinkedIn. The link was also sent by email to all current and past students (alumni), faculty and staff of the Lebanese American university (LAU). The data collection was approved by the LAU Institutional Review Board.

**Fig 1:**
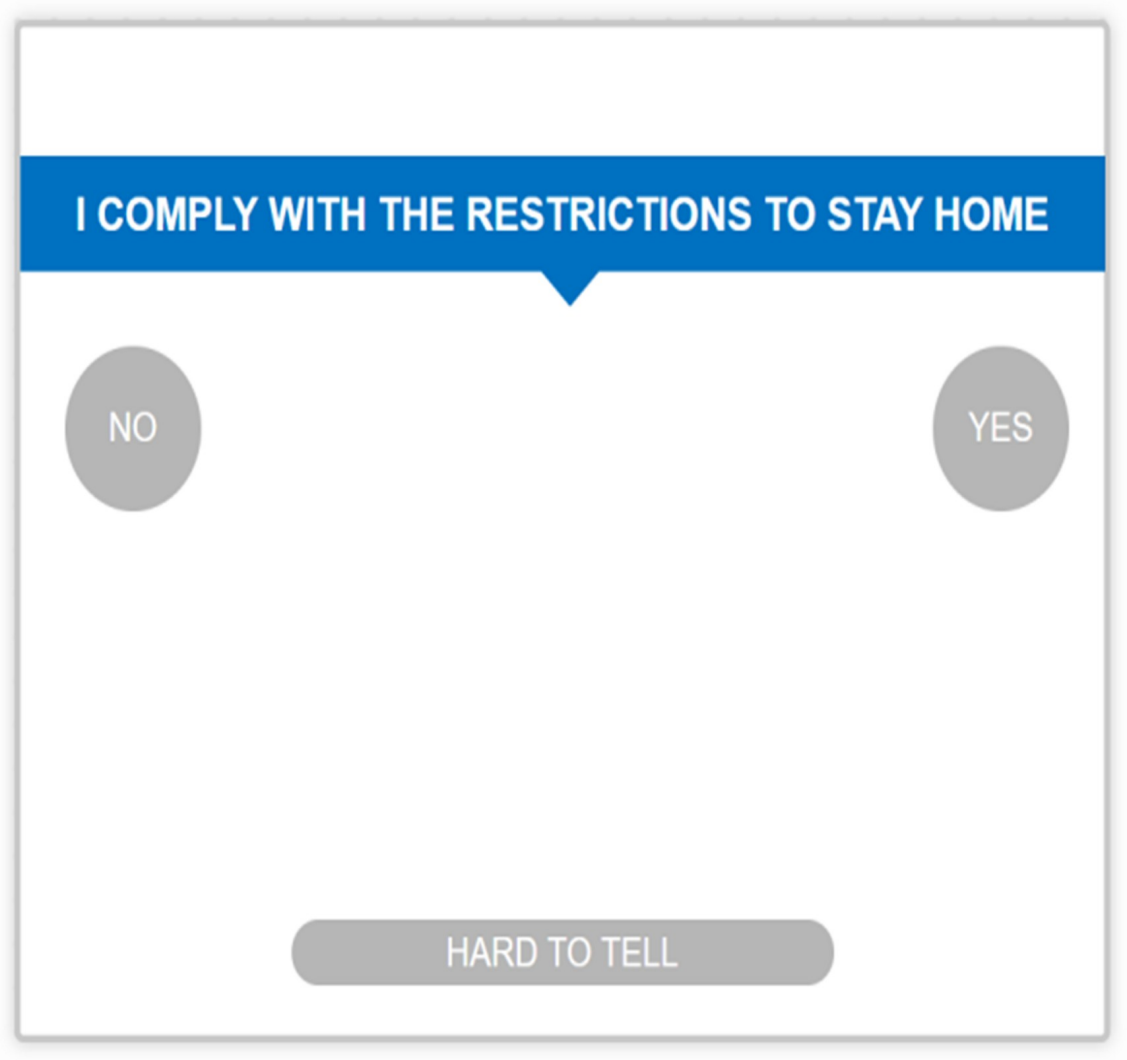
Test screen.

A series of statements were provided to respondents and their task was to agree with the sentence that appears on the screen. As shown in Fig 1, three possible answers were available: "YES", "HARD to TELL" and "NO". Simultaneously, the explicit and implicit responses were recorded, where implicit responses are reflected by reaction speed.

Before each statement, a control screen (Advanced Button) was introduced to ensure that the measured response time (RT) is not biased by the mouse’s position. More specifically, it forces the mouse position to maintain both yes and no responses at the same distance.

A calibration phase, which consisted of three parts, preceded the test part: (i) *Scale familiarization*. The purpose of this section was to familiarize respondents with the scale of responses and the position of the buttons on the screen; (ii) *familiarization with the purpose of the task and motor warm-up*. A series of statements explaining the purpose of the task were described. Participants were informed that opinions and certitude were measured by the test. Twelve such screens were presented in the main part of the test, introducing participants to the task; (iii) *Increasing the engagement of participants in the task*. A validation box appears twice to ensure that respondents pay attention to the presented statements, asking them to indicate what statement was shown on the previous screen. If the answer was incorrect, a message will show up asking for more careful work ("Please work carefully").

### Measures

Voicing opinions on sensitive issues is subject to distortions, especially when it involves expressing attitude and describing behavior. Declarative or explicit responses are often affected by political correctness, post rationalizations and auto presentation needs. According to Greenwald, and Banaji (1995), self-reported measures are insufficient to capture implicit processes, and thus indirect tools are required to enhance the accuracy of the explicit responses [32]. Most of such tools use respondents’ response time or reaction time as an indicator reflecting implicit processes [26]. The true revival of response time measurement occurred in the late twentieth century, when computers that could precisely measure tasks in milliseconds became more widely available, and the priming paradigm swept through academia [33].

The RT measurement used in this study follows the approach of Fazio’s theory of attitude accessibility [34]. Explicit opinion and implicit accessibility of attitude are two dimensions of attitudes [35]. Stronger attitudes are more accessible and therefore expressed with a shorter response time [28, 34]. Thus, the response time measurement provides results which are less affected by self-presentation bias or social desirability than conventional questionnaires [36, 37].

There were 56 statements in total in the survey. We identified 18 statements (Table 2) related to health worries, life satisfaction, social well-being concerns, and good COVID-19 practice. Responses to the statements were converted to a 4-likert scale based on the response confidence level. The resulting categories were as follows: 1 = “High NO”, 2 = “Low NO”, 3 = “Low YES”, and 4 = “High YES”.

In this study, COVID-19 health worries related statements are derived from the Coronavirus Health Impact Survey (CRISIS) [38]. The CRISIS survey aimed to assess people’s health worries about themselves, their friends and family members being infected by the virus and it has been recently used by Liu et al. [39]. On the other hand, the three statements examining life satisfaction fit an adapted shorten version of the Multidimensional Students ‘Life Satisfaction Scale (MSLSS) introduced and used in previous studies [40, 41]. Since social isolation is negatively associated with well-being [42–44], we created a social well-being concerns variable with four items related to pandemic isolation. The COVID-19 practice statements match the items used by Ferdous et al. [29] and are in line with World Health Organization’s infection prevention and control measures for COVID-19 [45].

The popular measure of reliability Cronbach’s alpha is computed for assessing the internal consistency of the construct items. For life satisfaction and practice, Cronbach’s alpha values were 0.58 and 0.60, respectively. Cronbach’s alpha for health worries was 0.68, while it was 0.70 for social well-being concerns. A value between 0.6 and 0.7 suggests an acceptable level of reliability [46]. Consequently, we generated scores for each construct by computing the average of its individual scale item scores. In addition to those outcomes, the survey included questions related to sociodemographic factors such as gender, income, age, education, living status, number of children, employment status, and city size. Summary statistics of the variables considered in this study are presented in the result section.

### Data preparation for analysis

The RT is measured in milliseconds (ms). Responses with a short RT (below 500 ms) are suspected of being given at random, while those with a long RT (above 10,000 ms) are suspected to be given after distraction [47]. This type of dubious responses that accounted for less than 1% of the total number of participants was deleted. To account for individual differences in reaction time, milliseconds were converted to natural logarithms [48] and the individual values are standardized into a scale from 0 to 1000 [25]. This standardized scale scores are automatically generated by iCode. A lower score indicates slower reaction, while faster reactions are reflected in higher scores. A cut-off score of 600 is used to identify high confidence in the participant’s response [25]. Furthermore, we have tried several confidence cut-offs between 600 and 1000 in our analysis and no significant change in the results was found.

### Statistical analysis

Descriptive statistics such as proportions and frequencies are computed. The dependent variables in this study (health worries, social well-being concerns, life satisfaction) are considered numerical. Therefore, we performed ordinary least squares (OLS) regression to study their associations with the demographic indicators. However, the constant-variance assumption (homoscedasticity) was violated. Thus, robust standard errors method is employed to correct heteroscedasticity. Using the variance inflation factor (VIF), we checked for multicollinearity among the predictors. The lowest and highest VIF values were 1.05 and 2.90, respectively. As a rule of thumb, a VIF value of at least 10 indicates the presence of serious multicollinearity [49]. Thus, no serious multicollinearity issues have been identified. As for COVID-19 good practice, we estimated a logistic regression. The internal consistency of the construct items was assessed based on Cronbach’s alpha. We run the analysis with R language (Version 3.4.4). Statistical significance was set at p-value <0.05.

## Results

### Descriptive statistics

The sociodemographic characteristics of the participants are reported in Table 1. Participants from various age groups, education levels and income classes were part of our sample. The median revenue class was between 1,000,000 L.L. and 2,500,000 L.L. Nearly two-thirds of the participants were women, with the majority (62%) between the ages of 18 and 25. The largest number of participants (90%) received a bachelor’s or higher degree or continue to study at university, with one third living in large cities (more than 100 000 inhabitants). Couples with children made up 23% of the sample. As for employment status, 34% were employed, 48% were students and 12% were unemployed.

**Table 1:** Sociodemographic characteristics of the participants.

Variable	n (%)
**Age (years)**	
18–25	589 (59.6)
26–35	197 (19.9)
36–49	133 (13.5)
50–64	46 (4.7)
≥65	9 (0.9)
Missing	14(1.4)
**Gender**	
Men	352 (35.6)
Women	609(61.6)
Missing	27(2.7)
**Education Level**	
Elementary school	8 (0.8)
Brevet degree or 9th grade	9 (0.9)
BACC II or Highschool	60 (6.1)
Vocational	33 (3.3)
Bachelor’s degree or higher	878 (88.9)
**Living status**	
Living alone	90 (9.1)
Living alone with children	57 (5.8)
Couple (married, or not) without children	42 (4.3)
Couple with children	226 (22.9)
Other	573 (58.0)
**Children**	
No	720 (72.9)
≥1	268 (27.1)
**City Size (people)**	
< 10,000	291 (29.5)
Between 10,000–14,999	158 (16.0)
Between 15,000–9,9999	67 (6.8)
Between 20,000–49,999	72 (7.3)
Between 50,000–99,999	71 (7.2)
≥100,000	329 (33.3)
**Employment Status**	
Employed	339 (34.3)
Student	469 (47.5)
Entrepreneur	48 (4.9)
Retired	9 (0.9)
Homemaker	3 (0.3)
Unemployed	120 (12.1)
**Monthly Income (LBP**^**‡**^**)**	
≤ 600,000	108 (10.9)
Between 600,000 L.L- 1,000,000	101 (10.2)
Between 1,000,000–2.500.000	171 (17.3)
Between 2,500,000–5,500,000	94 (9.5)
Between 5,500,000–8,500,000	59 (6.0)
≥8,500,000	34 (3.4)
Missing	421(42.6)

Note.

^‡^LBP is Lebanese pound

For each construct statement, the percentage and frequency of responses in terms of confidence level are reported in Table 2. Regarding health worries, participants demonstrated that the health of their older family members is their most worrying issue (94.8% Yes and High Yes). The social well-being concerns were more pronounced in respondents who are worried about not being able to meet with friends (62.5% Yes and High Yes). While Lebanese exhibited high satisfaction with their families (93.1% Yes and High Yes), their highest dissatisfaction was with their country (63% No and High No). As for COVID-19 practices, following the recommendations for social distancing was the most prevalent practice (93.1% Yes and High Yes).

**Table 2:** Mental well-being outcomes and COVID-19 practices among participants.

Variables	High No n (%)	No n (%)	Yes n (%)	High Yes n (%)
**Health worries**				
I’m afraid of becoming infected with coronavirus	217(24.1)	113(12.6)	176(19.6)	394(43.8)
I’m worried about my own health	174(18.5)	173(18.3)	202(20.4)	394(41.8)
I’m worried about the health of my children	207(25.7)	129(16.0)	150(18.6)	321(39.8)
I’m worried about the health of my older family members	16(1.7)	34(3.5)	260(27.0)	653(67.8)
**Social well-being concerns**				
I’m worried about not being able to meet with friends	105(11.5)	239(26.1)	288(31.4)	285(31.1)
I’m worried about not being able to meet with my family	126(13.4)	280(29.9)	317(33.8)	215(22.9)
I’m worried that living in isolation will negatively affect my well-being	120(13.5)	265(29.8)	274(30.8)	231(26.0)
I’m afraid that life in isolation will negatively impact my health	162(18.1)	259(29.0)	229(25.6)	244(27.3)
**Life satisfaction**				
I’m satisfied with my country	352(40.1)	201(22.9)	129(14.7)	195(22.2)
I’m satisfied with my family	30(3.2)	35(3.7)	208(22.1)	669(71.0)
I’m satisfied with myself	75(8.3)	62(6.8)	166(18.3)	604(66.6)
**Practice**				
I wash hands for a min. 30 seconds	68(7.4)	253(27.7)	398(43.5)	196(21.4)
I follow the government’s recommendations related to the pandemic	20(2.1)	28(3.0)	363(38.3)	537(56.6)
I try not to leave the house	59(6.1)	86(8.9)	374(38.9)	442(46.0)
I’m going to exercise regularly	105(13.3)	83(10.5)	204(25.8)	398(50.4)
I’m going to eat healthier	58(6.7)	56(6.5)	229(26.5)	520(60.3)
I follow the recommendations for social distancing	37(3.9)	27(2.9)	175(18.6)	700(74.5)
I motivate others to follow the recommendations related to the pandemic	16(1.7)	39(4.1)	434(45.5)	464(48.7)

### Health worries

Table 3 indicates that education is a significant predictor of health worries (β = -0.147, p < 0.01). People with higher level of education tend to be less worried about their health. Gender also shows a strong association with health worries (β = 0.176, p = 0.017), where men seem to be more worried than women. Additionally, people who have children experience more worry than those who do not (β = 0.218, p = 0.049). While age appears to be negatively associated with health worries, the statistical significance (p = 0.07) for such a conclusion is marginal. No significant associations were detected with the remaining demographic variables except for retirees in the employment status. However, the small number of retirees in the data is insufficient to make inference.

**Table 3:** Linear regression models for mental well-being outcomes in terms of sociodemographic factors.

Variables	Health worries		Social well-being		Life satisfaction	
	β (SE)	p-value	β (SE)	p-value	β (SE)	p-value
**Age**^**‡**^	-0.092(0.052)	0.070	-0.100(0.050)	0.046*	-0.074(0.042)	0.079
**Gender**^**†**^	0.176(0.074)	0.017*	0.123(0.074)	0.098	-0.058(0.065)	0.374
**Education**	-0.147(0.055)	0.009*	-0.064(0.066)	0.332	-0.072(0.047)	0.132
**Children**	0.218(0.111)	0.049*	-0.011(0.121)	0.930	0.285(0.093)	0.002*
**City Size**	0.009(0.018)	0.621	0.011(0.018)	0.533	-0.0181(0.015)	0.819
**Monthly Income**	-0.006(0.026)	0.83	0.000(0.027)	1.000	0.012(0.022)	0.416
**Employment Status**^**§**^						
Employed	0.099(0.144)	0.492	-0.030(0.141)	0.834	-0.029(0.114)	0.794
Student	0.09(0.154)	0.56	0.117(0.147)	0.425	-0.059(0.121)	0.624
Entrepreneur	0.186(0.203)	0.36	0.002(0.225)	0.993	0.089(0.164)	0.586
Retired	0.537(0.263)	0.042*	0.325(0.280)	0.247	-0.091(0.292)	0.754
Homemaker	----	----	----	----	0.663(0.124)	<0.001*
**Living Status** ^**¥**^						
Living alone	0.065(0.119)	0.586	0.225(0.113)	0.048*	0.236(0.086)	0.007*
Living alone with children	-0.095(0.171)	0.577	0.002(0.151)	0.990	-0.208(0.138)	0.133
Couple (married, or not) without children	0.117(0.153)	0.447	0.150(0.184)	0.416	-0.004(0.139)	0.973
Couple with children	0.01(0.115)	0.934	-0.035(0.120)	0.770	0.048(0.096)	0.614

Note. β Beta(s) are regression coefficients; SE standard errors are shown in parentheses, p is the p-value

‡ Youth groups (18–25) was used as a reference for Age

† Woman was used as a reference group for Gender

§ Unemployed was used as a reference group for Employment Status

¥ Other (living with parents or siblings) was used as a reference for Living Status

* p-value is significant (<0.05)

### Social well-being concerns

Individuals who live alone have significantly higher social well-being concerns than those who live with their parents or siblings (β = 0.225, p = 0.048). Younger people are more concerned about their social well-being during the pandemic (β = 0.1, p = 0.046). Gender was not significant, and the remaining sociodemographic predictors did not seem to have an effect on people’s social well-being (p > 0.05).

### Life satisfaction

We believe that lockdown and strict restrictions due to COVID-19 would affect individual’s life satisfaction. There is a significant association between living status and life satisfaction as there is a significant difference between the two categories "living alone" and “others” (p = 0.007). The difference in life satisfaction among the employment status categories was significant. The category “Home makers” in the employment status exhibit a significant difference in life satisfaction compared to unemployed individuals. However, this group had a very small number of respondents. Moreover, a significant association between life satisfaction and having children is detected (p = 0.002).

### Practice

The practice score variable was generated by computing the average scores of the responses to practice statements. For each question of practice, values of 3 and 4 indicate that the participant responded with “Yes” and “High Yes” for good practices, respectively. We used a cut-off of 3 for good practices and we estimated a logistic regression model with a dependent variable assuming a value of 1 if the practice score is at least 3 and zero otherwise. On the other hand, we combined the age categories into two groups to highlight differences in good practice between youth (18–25) and older groups (> 25). The estimated coefficients with their statistical significance, odds ratios, and 95% confidence intervals for odds ratios are reported in Table 4. As the table shows, age and having children were significant predictors for the good practice (p < 0.05). The youth group is set as a reference category for age and both predictors have positive coefficients. This demonstrates that older adults and individuals with children are more likely to have good COVID-19 practice than young people and those without children, respectively. After adjusting for the other socio-demographic variables, the odds of having good practices for older adults are 3.13 times higher than that of youth, while the odds for those with children are 3.18 times higher than those without.

**Table 4:** Logistic regression analysis for COVID-19 practice.

	β (SE)	OR	95% Confidence Interval		P-value
**Age**^**‡**^	1.142 (0.430)	3.132	[1.347; 7.278]		0.008*
**Gender**^**†**^	0.381 (0.335)	1.463	[0.758; 2.824]		0.256
**Education**	-0.023 (0.371)	0.977	[0.472; 2.023]		0.950
**Children**	1.157 (0.571)	3.181	[1.039; 9.742]		0.043*
**City size**	-0.109 (0.079)	0.897	[0.768; 1.047]		0.168
**Monthly Income**	-0.138 (0.117)	3.132	[0.692; 1.096]		0.240
**Employment Status**^§^					
Employed	0.734 (0.537)	0.871	[0.728; 5.968]		0.171
Student	0.853 (0.537)	2.084	[0.819; 6.726]		0.112
Entrepreneur	-0.387 (0.729)	2.346	[0.163; 2.831]		0.595
Retired	----	----	----		----
**Living Status** ^**¥**^					
Living alone	0.415 (0.537)	1.515	[0.529; 4.341]		0.439
Living alone with children	-0.383 (0.539)	0.682	[0.237; 1.963]		0.478
Couple (married, or not) without children	0.982 (1.079)	2.671	[0.323; 22.115]		0.362
Couple with children	-0.806 (0.545)	0.447	[0.153; 1.301]		0.140

Note. β Beta(s) are regression coefficients; SE standard errors are shown in parentheses, p is the p-value

‡ Youth groups (18–25) was used as a reference for Age

† Woman was used as a reference group for Gender

§ Unemployed was used as a reference group for Employment Status

¥ Other (living with parents or siblings) was used as a reference for Living Status

* p-value is significant (<0.05)

## Discussion

The COVID-19 pandemic has posed a range of threats to the general population worldwide and has affected public health at various levels. The primary goal of this study is to investigate health worries, social well-being, and life satisfaction in the Lebanese population during COVID-19 pandemic and their associations with sociodemographic factors. Additionally, we identified potential predictors of COVID-19 good practices. Lebanon is considered a high incidence area and little or no research has been published on this topic in the Lebanese context. Findings indicate that these mental well-being outcomes and practices are influenced by several socio-demographical factors.

Our data provided insight into some of the demographic factors associated with rising concern about health. A lower level of education was found to be associated with higher health worries. This finding is consistent with a cross-national study conducted in Norway, the United Kingdom, the United States, and Australia, which found that having a lower education was linked to more health worries and poor mental health [50]. According to the literature, a higher educational degree tends to be protective against anxiety and depression, which builds up over a lifetime [51]. Besides, the result that men have more health worries than women contradicts the findings of similar studies conducted during COVID-19 pandemic from various countries, which showed that women have more psychological worries, depression, and anxiety [12, 52, 53]. Lebanon, however, is a patriarchal society [54] in which men are the household’s principal financial providers. Since COVID-19 pandemic, combined with other crises, shut down many businesses in Lebanon, Lebanese men tend to feel more threatened and desperate if they lose their work and healthcare insurance coverage, which could heighten their health worries. Moving forward, we noticed greater health worries among individuals having children and being married. This result is consistent with the literature, which shows that parents with children have more worries and concerns, according to a recent study in Croatia [55].

The pandemic of COVID-19 has dramatically influenced interpersonal relationships [56]. Nevertheless, while the length of the pandemic cannot be anticipated, we are aware of the severe effect of these steps on society, on relationships and interactions between people, thereby impacting their social well-being. Age and living status have been shown to be strongly associated with social well-being issues. Young people have been more concerned about their social well-being since the pandemic. This is well linked with a recent comparative study, which found that younger age groups are more concerned than older groups about their social and psychological well-being [57]. Consistent with previous studies [58, 59], individuals who live alone exhibit a significantly higher social well-being concerns compared to those living with family members. Literature shows that being married/living together, or living with a larger number of adults, and having social support are considered to be protective factors [60].

A prominent characteristic of happiness is life satisfaction [61]. Our data suggests that while Lebanese are very satisfied with their families, their greatest dissatisfaction has been with their country. According to the world happiness index, Lebanon is ranked 91^st^, and this could be due to the worsened crises (financial, economic, unemployment, and health) that the nation has been facing recently [62], leaving Lebanese unhappy and dissatisfied with their country. In response to COVID-19, a study on social participation and people’s life satisfaction showed that keeping in touch with friends and family maintains an appropriate level of life satisfaction [63]. Our results revealed a significant association between the living status and life satisfaction. On the other hand, having children was associated with higher levels of life satisfaction. With respect to COVID-19 practices in Lebanon, the most prevalent practice was to follow the recommendations for social distancing. Continued evidence has shown that mask wear is one of the main preventive measures for stopping the spread of the infection in the community, in addition to social distancing, and washing hands with water and soap [11, 29, 64]. Moreover, our data displays that age and having children were significant predictors for good practices. After adjusting for the other socio-demographic variables, the odds of having good practices for older adults (age >25) were shown to be three times higher than that of the youth. Likewise, several studies have recently found that older age groups were associated wth good practices [29, 30, 65, 66]. Furthermore, the odds of having good practices for participants with children were three times higher compared to those without. The fact that children can be considered silent carriers of this highly infectious virus [67] may justify the need for families to follow good practices to protect vulnerable household members.

## Limitations

This study comes with some limitations. First, the survey was conducted online where respondents were recruited via social media channels. Therefore, the sample was collected conveniently and not randomly. However, as previously mentioned in the methodology section, convenience sampling has been extensively used in COVID-19 related studies. While this sampling technique cannot always ensure the generalizability of results, it can remain an efficient method for assessing the likelihood of potential relationships between variables [68–70]. Moreover, online surveys are disadvantageous to digital illiterates and to those who cannot afford internet access. However, to prevent possible virus transmission, online surveys remain relevant data collection tools during the pandemic.

Second, the low frequency representation in the sample of some groups, such as retirees and homemakers was also a constraint. The very low proportions of these groups prevent us from concluding them confidently, and hence related results should be treated with attention. Third, while our sample is considered large enough, the majority (60%) of respondents were below 25. This may reduce the statistical significance of age for some outcomes, such as life satisfaction. However, because Lebanon is a young society with a higher percentage of youth than the global average [71], our sample’s representativeness can be compromised. Around 80% of young adults (18–35 years) were involved in this study. This age group accounts for a sizable proportion (32%) of the total Lebanese population [72]. On the other hand, 98% of active internet users in the Mena region [73] are young adults. As a result, it is safe to assume that our sample is representative of the population of internet-literate Lebanese young adults, and that our findings apply to this population.

COVID-19 has posed new challenges to humanity and to people’s quality of life. Further research is therefore needed to understand its long-term impact on the individuals’ mental health. Lebanon has recently faced one of the largest non-nuclear explosions in history. Beirut blast that occurred on 4 August 2020 killed 220 people and left more than 6,500 injured and some 300,000 homeless. We believe that the effects of COVID-19 on the mental well-being of Lebanese would interact with those of the blast. Hence, a new data collection is required to test this hypothesis through a longitudinal study. It is important to note that the findings of the current study should be viewed as suggestive rather than conclusive, given the highlighted limitations, particularly the use of a convenience sample.

## Conclusion

This study intended to describe overall patterns in mental well-being outcomes and investigate their association with sociodemographic characteristics in the Lebanese society in response to COVID-19 pandemic. Our findings revealed that internet-literate Lebanese young adults in Lebanon showed significant differences in health worries, social well-being concerns, and life satisfaction across various sociodemographic factors. The suggestive findings of this study could pave the way for a well-coordinated national strategy and increased collaboration with public health practitioners to mitigate the pandemic’s adverse effects on mental health in the long-term.
